# New Bacteriophage *Pseudomonas* Phage Ka2 from a Tributary Stream of Lake Baikal

**DOI:** 10.3390/v17020189

**Published:** 2025-01-29

**Authors:** Valeriya Ilyina, Alina Gatina, Elena Trizna, Maria Siniagina, Liudmila Yadykova, Anastasiya Ivannikova, Georgiy Ozhegov, Daria Zhuravleva, Marina Fedorova, Anna Gorshkova, Peter Evseev, Valentin Drucker, Mikhail Bogachev, Shamil Validov, Maya Kharitonova, Airat Kayumov

**Affiliations:** 1 Institute of Fundamental Biology and Medicine, Kazan Federal University, 420012 Kazan, Russia; v_nikolayevna@inbox.ru (V.I.); alinka.zam@mail.ru (A.G.); trizna91@mail.ru (E.T.); marias25@mail.ru (M.S.); milayesyad@gmail.com (L.Y.); vanndkova@mail.ru (A.I.); georgii_provisor@mail.ru (G.O.); darya.ed@gmail.com (D.Z.); masfedorova97@mail.ru (M.F.); maya_kharitonova@mail.ru (M.K.); 2 Limnological Institute of the Siberian Branch of the Russian Academy of Sciences, 664033 Irkutsk, Russia; kovadlo@yandex.ru (A.G.); drucker@lin.irk.ru (V.D.); 3 Laboratory of Molecular Microbiology, Pirogov Russian National Research Medical University, 117997 Moscow, Russia; petevseev@gmail.com; 4 Biomedical Engineering Research Centre, St. Petersburg Electrotechnical University, 197022 St. Petersburg, Russia; rogex@yandex.com; 5 Laboratory of Molecular Genetics and Microbiology Methods, Kazan Scientific Center of the Russian Academy of Sciences, 420111 Kazan, Russia; sh.validov@knc.ru

**Keywords:** bacteriophage, *Pseudomonas aeruginosa*, antimicrobial resistance, synergistic effects

## Abstract

*Pseudomonas aeruginosa*, an opportunistic pathogen, causes various biofilm-associated infections like pneumonia, infections in cystic fibrosis patients, and urinary tract and burn infections with high morbidity and mortality, as well as low treatment efficacy due to the extremely wide spread of isolates with multidrug resistance. Here, we report the new bacteriophage *Pseudomonas* phage Ka2 isolated from a tributary stream of Lake Baikal and belonging to the *Pbunavirus* genus. Transmission electron microscopy resolved that *Pseudomonas* phage Ka2 has a capsid of 57 ± 9 nm and a contractile and inflexible tail of 115 ± 10 nm in the non-contracted state. The genome consists of 66,310 bp with a GC content of 55% and contains 96 coding sequences. Among them, 52 encode proteins have known functions, and none of them are potentially associated with lysogeny. The bacteriophage lyses 21 of 30 *P. aeruginosa* clinical isolates and decreases the MIC of amikacin, gentamicin, and cefepime up to 16-fold and the MIC of colistin up to 32-fold. When treating the biofilms with Ka2, the biomass was reduced by twice, and up to a 32-fold decrease in the antibiotics MBC against biofilm-embedded cells was achieved by the combination of Ka2 with cefepime for the PAO1 strain, along with a decrease of up to 16-fold with either amikacin or colistin for clinical isolates. Taken together, these data characterize the new *Pseudomonas* phage Ka2 as a promising tool for the combined treatment of infections associated with *P. aeruginosa* biofilms.

## 1. Introduction

*Pseudomonas aeruginosa*, a gram-negative rod-shaped bacterium, is widely spread in various niches such as water, soil, plants, swimming pools, and sanitary equipment and colonizes the mucous membrane of the oral cavity and skin of humans [1,2,3,4,5]. In immunocompromised patients, it causes acute and chronic infections such as ventilator-associated pneumonia, otitis, urinary tract and bloodstream infections, burn wounds, and keratitis [6], with a mortality rate of up to 40%. Moreover, *P. aeruginosa* is the main cause of sepsis in cancer patients, the first cause of respiratory failure and nosocomial pneumonia [7]. Due to its high intrinsic and acquired resistance to antibiotics, *P. aeruginosa* has been classified as a multidrug-resistant (MDR) member of the ESKAPE group of bacteria [8]. Therefore, the number of available tools for targeting gram-negative bacteria, including *P. aeruginosa*, decreases and thus challenges the development of new approaches [9,10].

One of the intrinsic mechanisms of antibiotic resistance in *P. aeruginosa* involves the formation of a biofilm, a community of microorganisms bound to the surface and embedded into the extracellular matrix [11]. Biofilms are formed on both body tissues and invasive devices such as ventilators, endotracheal tubes, and catheters [12]. While in biofilms, bacteria become up to 1000 times more resistant to antibiotics compared to free-living cells due to the diffusion barrier of the matrix [13]. Many investigations have shown that biofilm formation by *P. aeruginosa* significantly complicates the treatment of infections; moreover, in some cases, the biofilm may lead to the death of the patients, for example, in the case of infections in cystic fibrosis patients (reviewed in [10,14]). Therefore, antimicrobial treatment must be adjusted to also include an antibiofilm strategy [15].

Various studies have shown the effectiveness of phage therapy without any noticeable side effects [16,17,18,19,20], including treating multidrug-resistant *P. aeruginosa* infection [21,22]. The effective use of phage therapy has been demonstrated in various animal models and clinical studies as a therapy for the treatment of *P. aeruginosa* infections, especially MDR and XDR [23]. For example, a single co-administration of bacteriophage and Ceftazidime resulted in the successful treatment of a *P. aeruginosa* vascular graft infection [7]. Nevertheless, the phages putatively offered for the treatment of bacterial infections should have no possible lysogenic pathway as they have been reported to increase bacterial resistance to antibiotics and other phages [24,25], and only lytic phages can be used due to their rapid reproduction, low toxicity, and destruction of bacterial biofilms [26,27].

Among various phages lysing *P. aeruginosa*, *Pbunoviruses* have been reported as efficient antibacterial tools. *Pbunavirus* is a genus of viruses in the family *Myoviridae* and, to date, includes up to 40 species of *Pseudomonas aeruginosa* viruses isolated from waters of North America [28], South America [29], Europe [30], Russia [31], and China [32]. Generally, phages of the genus *Pbunavirus* show a high lytic potential, infecting more than 30% of the tested *P. aeruginosa* isolates, and thus represent a promising tool for antibacterial therapy [31,33]. The efficacy of bacteriophages against *P. aeruginosa* biofilms has been reported [34,35], including the treatment of foot ulcers and purulent wounds [36,37] and as disinfectants in the food industry [38]. Bacteriophages can also inhibit *P. aeruginosa* biofilm formation by hydrolyzing acyl homoserine lactones and inhibiting quorum-sensing activity [22]. Moreover, the combination of bacteriophages with certain antibiotics has shown noticeable effectiveness in destroying *P. aeruginosa* biofilms [39].

In this study, we characterize the new bacteriophage *Pseudomonas* phage Ka2 isolated from a tributary stream of Lake Baikal, including its effect on various strains of *P. aeruginosa* in the planktonic and biofilm-embedded form as well as its combined effect with antimicrobial drugs.

## 2. Materials and Methods

### 2.1. Bacterial Strains and Growth Conditions

*Pseudomonas aeruginosa* PAO1 has been used as a host strain. A series of clinical isolates (isolates 1475, 293, 305, 449, 458, 278, 288, 398, 410, 443, 250, 4086-2023, 4241-2023, 3101, 2806, 185, 286, 206, 347, 369, 383, 400, 639, 13, 88, 99, 176, 465, 115, and 468) obtained from the Kazan Institute of Microbiology and Epidemiology, Kazan, Russia (see Table 1 for the resistance pattern and source) were used for specificity testing. Bacteria were grown and maintained in LB broth (g/L: tryptone—10, yeast extract—5, NaCl—5, and pH of 7.0; the 3 × LB contained all compounds in a three-fold higher amount). The basal medium (BM, g/L: peptone—7.0, MgSO_4_ × 7H_2_O—2.0, CaCl_2_ × 2H_2_O—0.05, and glucose—1%) was used for the biofilm assays [40].

### 2.2. Bacteriophage Isolation, Propagation, and Purification

*Pseudomonas* phage Ka2 was isolated from the water obtained from a tributary stream of Lake Baikal, close to Slyudyanka town, and deposited in the All-Russian Collection of Industrial Microorganisms (VKPM), ID F-1630. Water samples (100 mL) were *filtered through nitrocellulose* membranes (0.45 μm pore). Five mL of *Pseudomonas aeruginosa* PAO1 overnight culture and 10 mL of 10 × LB were added to 85 mL of filtrate, and 1 × LB was used for inoculation as a cell growth control. The enrichment and control cultures were incubated at 37 °C for 24 h in an orbital shaker. From the enrichment cultures where clarification was observed compared to the control, indicating possible lysis by bacteriophages, a 1 mL aliquot was taken and mixed with chloroform (30 µL), followed by incubation for 30 min at 4 °C. The suspension was centrifuged (10 min, 1700× *g*), and the supernatant was titrated to obtain a series of 10-fold dilutions in sterile 0.9% NaCl. From each dilution, 10 µL were dropped on double-layer LB agar plates containing 10^7^ CFUs/mL of *P.* aeruginosa PAO1 in 0.75% top agar. Individual plaques were picked and propagated in double-layer agar plates two times in order to purify the phages.

### 2.3. PFU Count and Assessment of Bacterial Susceptibility to Phage

The PFUs of bacteriophage were counted using the Gratia assay (Gratia, 1936), with modifications [41]. Briefly, 90 µL of 0.9% NaCl was added to a sterile 96-well plate in 10 consecutive wells. Next, 10 µL of phage lysate was added to the first well, and serial 10-fold dilutions of phage lysate in sterile saline solution were prepared until 10^12^ dilution-fold. After that, 10 µL of suspension from each well was added to 1 mL of semi-liquid LB agar containing *P. aeruginosa* (10^7^ CFUs/mL) and loaded onto the surface of LB agar in Petri plates. The plates were incubated for 24 h at 37 °C, and PFUs were counted from dishes containing at least five plaques and multiplied by the dilution factor.

The bacterial susceptibility to phage was tested using a spot test [42] by dropping 5 µL of phage lysate (10^8^ PFUs/mL) onto plates covered with double-layer agar. For that, LB agar was coated with 3 mL of semi-liquid LB agar containing 30 µL of *P. aeruginosa* culture (with a density of 10^7^ CFUs/mL). Then, the plates were dried for 15 min and incubated at 37 °C for 24 h. The sensitivity of bacteria to the phage was assessed on the following scale: pronounced lysis (++++) if a completely transparent spot, with no bacterial growth inside, was formed; (+++) excellent or (++) good lysis if non-complete transparent spots or isolated negative colonies of bacteriophages inside, respectively, were formed; weak lysis (+); and lack of activity (−) if no distinguishable spot could be observed with the naked eye.

### 2.4. Determination of Optimal Multiplicity of Infection (MOI) and One-Step Growth Assay

To determine the optimal multiplicity of infection, *P. aeruginosa* PAO1 cells in a logarithmic phase (OD600 = 0.4–0.5) were harvested, washed with sterile NaCl solution, and mixed with the bacteriophages to the desired MOI values (10, 1, 0.1, 0.01, and 0.001). The mixture of bacteriophages and bacteria was incubated at room temperature for 15 min to ensure the adsorption of bacteriophages onto bacterial cells, and then, the resulting mixture was cultured on a shaker at 28 °C and 180 rpm for 12 h. The final amount of viral particles was determined by a spot test of 10-fold dilution series, and the optimal multiplicity of infection (OMOI) was calculated.

The one-step growth curve experiment was performed as described in [43], with modifications. Briefly, *P. aeruginosa* PAO1 cells in the middle exponential growth phase were harvested from 5 mL of culture (OD600 = ~0.3), washed by NaCl solution (0.9%), and resuspended in LB broth. Bacterial cells were mixed with bacteriophage solution until an MOI of 0.001 was obtained and were incubated for 10 min at 28 °C to ensure adsorption of the bacteriophage to the bacterial cell. Bacteria were separated from the free-floating bacteriophages by centrifugation (2 min, 9700× *g*) and resuspended in 10 mL of LB broth, followed by incubation at 28 °C with shaking at 180 rpm, and phage titers were determined at 10 min intervals by the double-layer agar approach. Bacteriophage titers at different time points were expressed as PFUs/mL, and a graph was plotted to create a one-step growth curve to determine the latent period and the burst size of the phage. The experiment was performed three times. The size of the spike was determined by counting the amount of bacteriophage titer at the end of the period divided by the number of infected bacteria (initial PFU count).

### 2.5. Impact of Serum on Bacterial Lysis

The efficiency of bacterial lysis in the presence of serum was determined as described in [44], with modifications. Briefly, 10 μL of the *P. aeruginosa* PAO1 culture with OD_600_ = 0.5 was diluted with 40 μL of LB in a 96-well plate. Then, 50 μL of either bacteriophage Ka2 suspension or sterile 0.9% NaCl solution was added to the wells, and incubation was followed for 2 h at 37 °C. Next, 100 μL of either active or inactive bovine serum (the serum was inactivated by incubating at 60 °C for 30 min) was added (thus making the final serum concentration 50%), and the plate was incubated for the next 2 h at 37 °C. Then, a ten-fold serial dilution of each sample was prepared, and the CFUs were counted.

### 2.6. Determination of Minimum Inhibitory Concentration (MIC)

The MIC of antimicrobials was determined by the broth microdilution method in 96-well microtiter plates according to the EUCAST rules for antimicrobial susceptibility testing [45]. The bacterial suspension containing 10^8^ CFUs/mL was subsequently diluted 1:300 with Mueller–Hinton broth, and antimicrobials were added to final concentrations: amikacin and meropenem in the range of 0.0078–8 µg/mL, gentamicin and colistin in the range of 0.00097–1 µg/mL, and ciprofloxacin in the range of 0.00048–0.5 µg/mL. The MIC was determined as the lowest concentration of the compound for which no bacterial growth could be observed after 24 h of incubation as assessed with the AlamarBlue (resazurin) test. The susceptibility of clinical isolates to antimicrobials was assessed by using the disk diffusion test, as recommended by EUCAST [45]. Briefly, disks of antimicrobials (Azlocillin (75 µg/disc), Aztreonam (30 µg/disc), amikacin (30 µg/disc), gentamicin (10 µg/disc), Levofloxacin (5 µg/disc), Piperacillin (100 µg/disk), Tobramycin (10 µg/disk), Ceftazidime (10 µg/disc), and ciprofloxacin (5 µg/disc) (NICP—Research Center for Pharmacotherapy, St. Petersburg) were placed onto LB agar inoculated with *P. aeruginosa* suspension (with density of 10^7^ CFUs/mL), and the plates were incubated for 24 h. The diameter of the growth inhibition zone was measured across the area in which growth was prominently reduced, and the results were interpreted according to available breakpoints as sensitive (S), resistant (R), or sensitive at increased exposure (I).

### 2.7. Biofilm Assays

*P. aeruginosa* PAO1 was inoculated in BM broth with the initial density of 2–9 × 10^6^ CFUs/mL) and grown in 24-well TC-treated polystyrol plates (1 mL per well) for 48 h under static conditions. Next, the broth was replaced by the new one, supplemented with antimicrobials in concentrations as indicated, phage Ka2 was added in experimental wells (10 µL of phage lysate with a titer of 10^8^ PFUs/mL), and incubation was performed for the next 24 h. Then, the culture liquid was removed, wells were washed with sterile phosphate-buffered saline (PBS) to remove nonadherent cells, and residual biofilms were analyzed with any crystal violet staining [46,47] for the total biofilm biomass evaluation, CFU count assay [48] with modifications [47], and AlamarBlue test to evaluate changes in the number of viable cells, SEM, or CLSM for the biofilm structure characterization.

### 2.8. Colony-Forming Unit Count

The viable cells (CFUs) were counted by using the drop plate assay as described in [48]. For that, wells were rinsed gently twice with 0.9% NaCl, and the biofilms were suspended in 0.9% NaCl by scratching the well bottoms with subsequent intensive pipetting to facilitate the disintegration of bacterial clumps. Next, a series of 10-fold dilutions of suspensions were prepared, and 5 µL of suspension was dropped onto LB plates. CFUs were counted from drops containing 5–10 colonies, averaged, and multiplied by the dilution factor.

### 2.9. Transmission Electron Microscopy (TEM)

The isolated bacteriophages were purified by picking up two clear plaques using a sterile tip. The tip was then placed in 100 µL of physiological saline (1 h at 4 °C), and the suspension was centrifuged (15 min, 1700× *g*) and sterilized through a 0.22 µm filter. The phage suspension was tittered, and a drop of phage (10^10^ PFUs/mL) was placed on a formvar-coated copper grid and stained with 2.0% (*w*/*v*) uranyl acetate. A transmission electron microscope (LEO 906E, Carl Zeiss, Jena, Germany), located at the Shared Research Facilities for Physical and Chemical Ultramicroanalysis LIN SB RAS, Limnological Institute, Irkutsk, was used for a detailed analysis of the phage sample.

### 2.10. Scanning Electron Microscopy (SEM)

The structure of the biofilms after treatment with *Pseudomonas* phage Ka2 was assessed with scanning electron microscopy. The biofilms were grown for 48 h in BM broth in 34 mm plastic adhesive Petri dishes (TC-treated, SPL Lifesciences, 3 mL per plate) under static conditions. Next, the broth was exchanged, and fresh BM broth containing *Pseudomonas* phage Ka2 was added. After three and six hours of incubation, the plates were washed three times with water and fixed with glutaraldehyde (1% water solution) for 24 h. After the subsequent washing with deionized water, the plates were dried for 12 h at 55 °C and coated in a vacuum with gold on an SCD 004 (Balzers AG, Balzers, Liechtenstein) and analyzed on a Quanta 200 microscope (FEI Company, Hillsboro, OR, USA) at 29 kV at the Shared Research Facilities for Physical and Chemical Ultramicroanalysis Research Center, Limnological Institute of the Siberian Branch of the Russian Academy of Sciences, Irkutsk.

### 2.11. Confocal Laser Scanning Microscopy

CLSM was performed using an Olympus IX83 inverted microscope (Olympus Europa, Hamburg, Germany) supplemented with a STEDYCON ultrawide extension platform. Biofilms to be analyzed were grown for 48 h in BM broth on cell imaging cover slips (Ibidi, Gräfelfing, Germany), treated with various antimicrobials at concentrations, as indicated, in the presence or absence of *Pseudomonas* phage Ka2 for 24 h. Then, biofilms were stained for 15 min with 3,3′-Dihexyloxacarbocyanine iodide at a final concentration of 0.02 µg/mL (green fluorescence) and propidium iodide (Sigma-Aldrich, St. Louis, MO, USA) at a final concentration of 3 µg/mL (red fluorescence) to differentiate between live and dead cells.

### 2.12. Sequencing of Pseudomonas Phage Ka2 Genome and Bioinformatics

The genomic DNA of *Pseudomonas* phage Ka2 was extracted from 3 mL of phage lysate by using an Invitrogen viral DNA extraction kit and sequenced on the Illumina MiSeq platform. DNA was sheared to fragments ranging between 300 and 500 bp using Covaris S220 (Covaris LLC, Woburn, MA, USA). The fragmented DNA sample was end-paired, dA-tailed, and ligated to the adaptor using the NEBNext Ultra II DNA Library prep kit for Illumina (NEB, Ipswich, MA, USA). The adaptor-ligated products were purified and further enriched using PCR, and paired-end sequencing was performed by using Illumina Miseq (Illumina, San Diego, CA, USA). Sequence read quality was assessed using FastQC (version 0.11.5) [49]; the genome was assembled using Unicycler 0.4.8-beta. The assembled genome was annotated using Prokka 1.14.5 [50] with subsequent manual verification by the alignment of predicted proteins with the Uniprot database and HHpred [51]. The annotated genomic sequence of *Pseudomonas* phage Ka2 has been deposited to NCBI, Bethesda, MA, USA GenBank and is available under accession number ON529291.

Protein structures were predicted using AlphaFold 3 (AlphaFold, AF) [52] and visualized with PyMOL 2.5.4 (Schrödinger Inc., New York, NY, USA). A search for similar structures was conducted using the DALI server [53]. The structural similarity was evaluated with the DALI Z-score [54].

The distribution of genes into subsystem categories was performed using the RAST server [55]. The genomic and protein alignments were performed using Blast Global Alignment [56]. The circular genome, distribution of ORFs, and GC content were visualized with SnapGene DNA (https://www.snapgene.com/snapgene-viewer, accessed on 20 September 2024) and Geneious Prime (https://www.geneious.com, accessed on 20 September 2024).

The nucleic and amino acid sequences of *Pseudomonas* bacteriophages were obtained from the NCBI database (https://www.ncbi.nlm.nih.gov/, accessed on 10 September 2024): ON375838.1, LC472884.1, FM201282.1, OP310975.1, NC_041870.1, LN610579.1, MT118298.1, MT119368.1, MT133562.1, NC_050145.1, MT119376.1, MT119363.1, MW595221.1, LC472883.1, LC102730.1, OM953790.1, MT118297.1, NC_041865.1, NC_042079.1, and ON857930.1. Average nucleotide identity (ANI) was calculated using FastANI [57]. For the phylogenetic analysis, amino acid sequences of the major capsid protein were used. The Virus Intergenomic Distance Calculator (VIRIDIC) tool, which offers an implementation of the traditional algorithm used by the International Committee on Taxonomy of Viruses (ICTV) and allows pairwise nucleotide identity to be calculated and compared using the BLAST tool, with several advantages [58], was used for the comparison of phage genomes. Sequence alignment was performed in MAFFT v7.490 using the L-INS-i algorithm [59]. The best substitution model (Q.pfam + G4) was obtained using IQ-TREE v2.2.6 ModelFinder [60], and the maximum likelihood phylogenetic tree was constructed using IQ-TREE v2.2.6 with standard bootstrap analysis (1000 replicates) [60]. Superimposition of protein structures was performed using PyMOL v3.

### 2.13. Statistics

All experiments were performed in three independent biological repeats with three technical repeats in each run. The statistical significance of the results was assessed using the Kruskal–Wallis statistical test with a significance threshold of *p* < 0.05. The fraction of viable cells on CLSM images and biofilm-covered areas on SEM images were quantified by using BioFilmAnalyzer v. 1.1, a software developed in-house [61].

## 3. Results

### 3.1. Specificity, Morphological Characteristics, and Lysis Kinetics of Ka2

**Figure 1:**
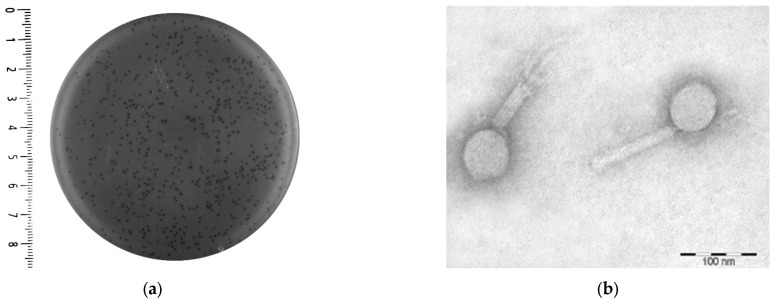
Morphology of *Pseudomonas* phage Ka2 plaques on a lawn of *P. aeruginosa* PAO1 after 24 h incubation (**a**) and transmission electron microscopy of *Pseudomonas* phage Ka2 virions (**b**). Virions were negatively stained with 2% (*w*/*v*) uranyl acetate. The scale bar is 100 nm (**b**).

*Pseudomonas* phage Ka2 was isolated from a tributary stream of Lake Baikal, close to Slyudyanka town. It forms round and pure plaques on the lawn of the host strain *P. aeruginosa* PAO1 (Figure 1a). In addition, Ka2 was able to lyse 21 of 30 clinical isolates (70%) of *P. aeruginosa*, including nine MDR isolates (Table 1), and no lysis of either *Pseudomonas* species (*P. putida*, *P. fluorescencs*, and *P. stutzeri*) or enterobacteria (*E. coli*, *K. pneumonia*, and *S. enterica* serovar Typhymurium) was observed, suggesting specificity of Ka2 to *P. aeruginosa*. Transmission electron microscopy revealed that Ka2 has a non-elongated capsid of approximately 57 ± 9 nm in diameter attached to a long, contractile, and nonflexible tail of approximately 115 ± 10 nm in length, thus giving a 172 ± 12 nm length for the whole virion (Figure 1b).

**Figure 2:**
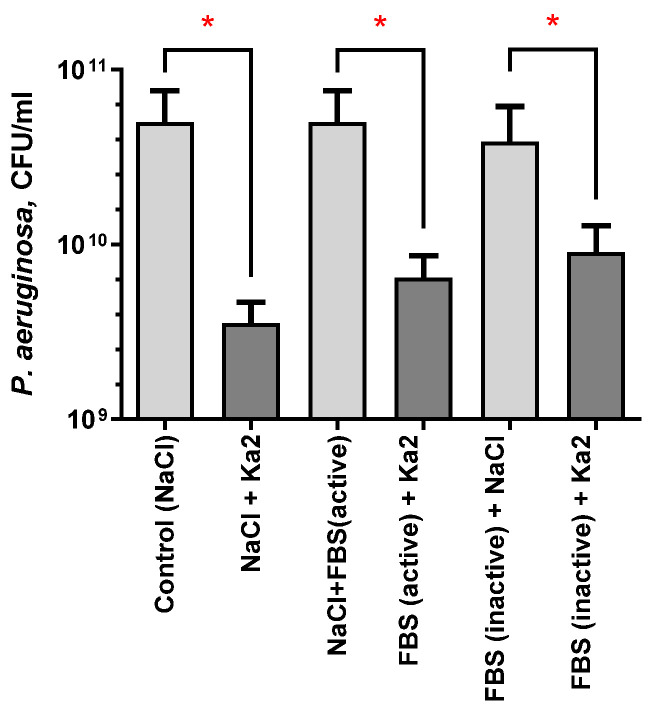
Impact of fetal bovine serum (FBS) on the lytic properties of bacteriophage Ka2. A sterile NaCl (0.9%) served as an added control instead of phage or FBS. Asterisks show statistically significant difference (Mann–Whitney U test, *p* < 0.05).

Similar to several studies that have reported the impact of serum on the lytic activity of phages [44,62], the lysis of *P. aeruginosa* PAO1 by Ka2 phage was examined in the presence of either active or heat-inactivated 50% fetal bovine serum. As can be seen from Figure 2, the presence of the serum does not impact the lytic activity of Ka2 since in all variants, a comparable rate of reduction in viable bacterial cells could be observed (1.2, 1.1, and 0.8 of log_10_ reduction).

**Figure 3:**
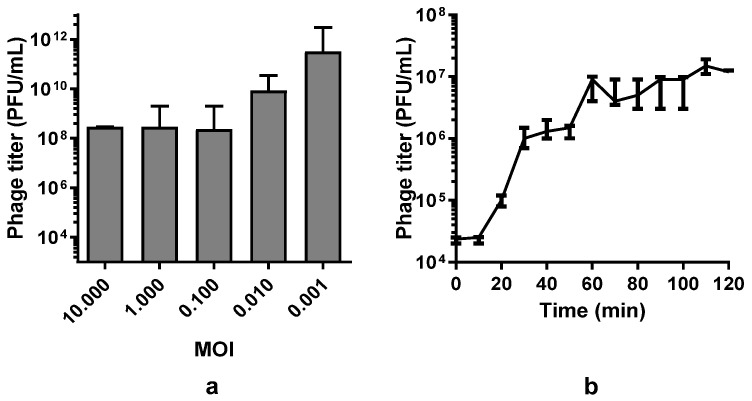
Determination of optimal MOI for bacteriophage Ka2 (**a**) and one-step growth curve (**b**). The phage titer was calculated at the indicated time points by using a double-layer overlay assay. Three independent experiments were performed. The data show the median with the range.

Next, the lysis kinetics of *Pseudomonas* phage Ka2 were investigated. The phage was able to infect *P. aeruginosa* PAO1 at various concentrations of viral particles, with the maximal lysis observed at a multiplicity of infection (MOI) of 0.001 (Figure 3a). The one-step growth assay revealed that the latent period of the bacteriophage Ka2 is about 10–20 min, with a burst size of Ka2 of approximately 300 PFUs/infected cell and a release time of 40 min (Figure 3b).

### 3.2. General Genome and Proteome Characterization

The whole genome of *Pseudomonas* phage Ka2 was sequenced using a high-throughput Illumina MiSeq platform with paired-end mode. The genome was assembled using Unicycler v. 0.4.8.0, resulting in a single circular contig with a length of 66,310 base pairs (bp) with an average coverage of 160× and GC content of 55%.

A BLASTn search performed using the NCBI nt database and the Ka2 genome as a query revealed a close relationship of Ka2 to phages assigned to the genus *Pbunavirus* (Table 2). Average nucleotide identity (ANI) calculations compared to phage Ka2 using FastANI and all 197 *Pbunavirus* phage genome sequences deposited in the database NCBI Genome identified *Pseudomonas* phage phiKTN6 (accession number KP340288.1), *Pseudomonas* phage vB_PaeM_FBPa50 (accession number ON375838.1), and *Pseudomonas* phage S50 (accession number LC472884.1) as having the highest ANI values (97.78%, 97.74%, and 97.24%, respectively).

**Figure 4:**
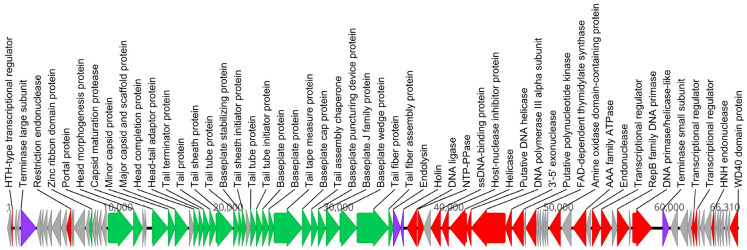
*Pseudomonas* phage Ka2 genome and functional mapping of genes. The direction of transcription is shown by arrows. Functionally assigned ORFs are highlighted based on their general functions: gray—hypothetical genes, green—structural genes, red—genes of replication, transcription, and processing of nucleic acids, and purple—genes of lysis.

The genome of Ka2 contains 96 predicted protein-coding genes, among which 52 (54.2%) were functionally assigned (Figure 4). Protein-coding genes were assigned to several functional groups based on predicted functions: 26 genes of structural proteins, 22 genes of replication, transcription, and processing of nucleic acids, and 4 genes of lysis. No genes of integrase, transposase, and recombinase or genes of regulators of lysogeny were found in the Ka2 genome.

**Figure 5:**
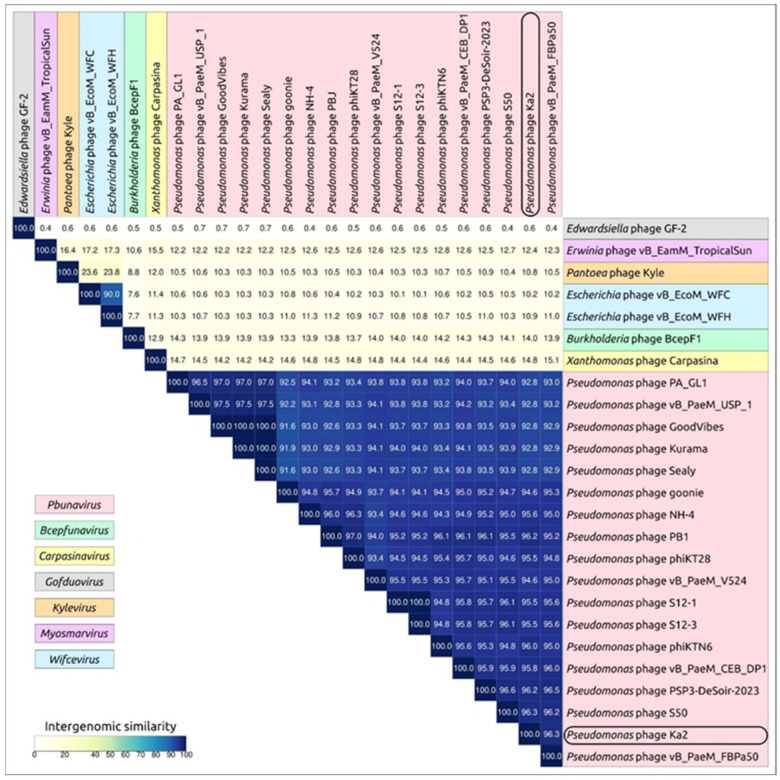
VIRIDIC-generated heatmap of 25 phages belonging to the *Pbunavirus* genus and related genera. The color coding indicates the clustering of the phage genomes based on intergenomic similarity. The numbers represent the similarity values for each genome pair, rounded to the first decimal. Phage taxonomy is indicated by a colored background, explained in the legends.

Intergenomic similarity between phage Ka2 and the related phages was calculated using the Virus Intergenomic Distance Calculator (VIRIDIC), recommended by the International Committee on Taxonomy of Viruses (ICTV). VIRIDIC calculations revealed a number of phages most similar to phage Ka2. The list of closest phages includes the phages found using the BLAST search mentioned above and other related phages. These phages show a high level of intergenomic similarity to phage Ka2 and several *Pbunavirus* phages, up to 96.3% over the entire genome length (Figure 5). This value significantly exceeds the intergenomic similarity threshold of 70% for genus delimitation set by the ICTV [63]. It is also slightly above the 95% threshold for species delineation, and strictly speaking, several isolated and published phages, including phages Ka2, phiKTN6, vB_PaeM_FBPa50, and S50, can be considered a clonal group of one specium.

**Figure 6:**
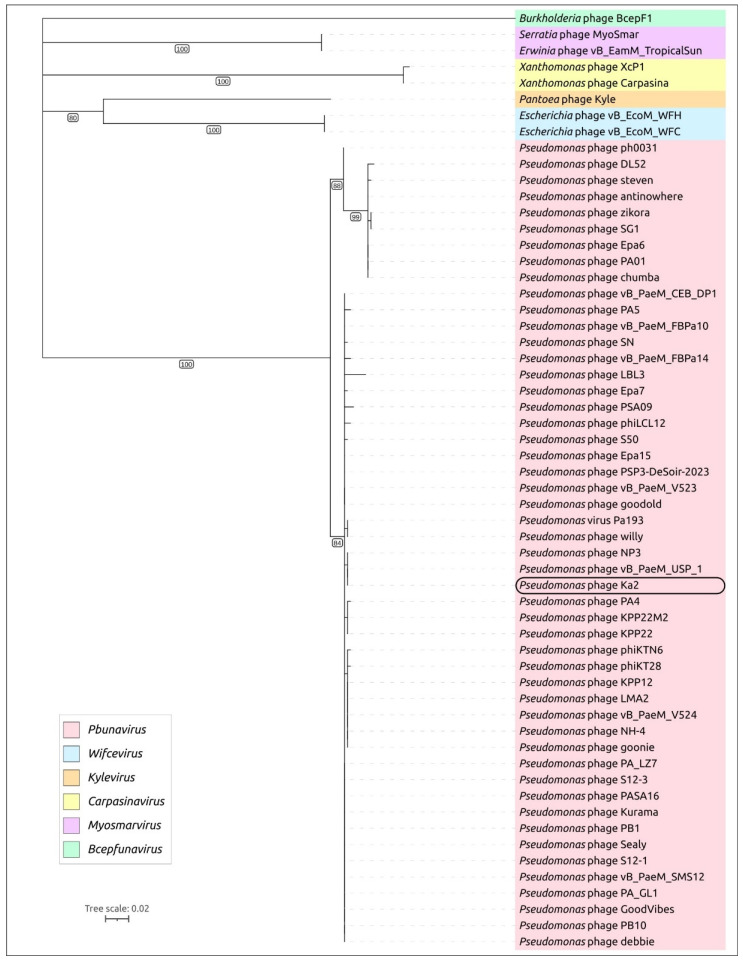
Maximum likelihood phylogenetic trees based on amino acid sequences of the major capsid protein of *Pseudomonas* phage Ka2 and related phages. The taxonomy of phages is indicated by a colored background, explained in the legends. Branches with a bootstrap support lower than 50% were deleted. Bootstrap values are shown near their branches. The scale bar shows 0.02 estimated substitutions per site, and the tree was unrooted.

The genes for the major capsid protein (MCP), large subunit terminase, and portal protein encode conserved proteins present in all tailed phages (class *Caudoviricetes*) [64] and can be used for taxonomic grouping purposes according to the ICTV’s recommendations [63]. To assess the phylogenetic position of phage Ka2, a BLAST search using the amino acid sequence of 50 MCPs, including the MCP of phage Ka2 and MCPs representing officially classified *Pbunavirus* species and related genera, was performed. The tree (Figure 6) placed phage Ka2 in a clade common with other *Pbunavirus* phages and distant from other related genera, indicating that phage Ka2 belongs to the genus *Pbunavirus*, consistent with the results of intergenomic similarity calculations and VIRIDIC clustering. Notably, phage Ka2 was placed in the same major subclade as phages PB1, phiKTN6, vB_PaeM_FBPa50, and S50.

The phage Ka2 adsorption apparatus has been predicted to include tail fibers. Typical phage tail fibers consist of three copies of tail fiber protein (TFP) [65,66]. The AlphaFold-predicted structure of the Ka2 tail fiber protein was used in a DALI search, which indicated a structural similarity between the Ka2 TFP trimer and tail fibers of other phages and pyocin fibers (Supplementary File S1), e.g., PDB structures #5NXF [67] and #6CL5 [68]. The predicted structure of the Ka2 tail fiber protein has an N-terminal α-helical domain (about 1–80 aa), apparently responsible for binding to phage particles (Supplementary Figure S1). Also, AlphaFold predicted a complex multi-domain structure of the Ka2 TFP (Supplementary Figure S2), which includes several central domains consisting mainly of β-sheets connected with linkers and the C-terminal part, reminiscent of carboxy-termini of other phage TFPs. At least seven central domains can be identified; they correspond to approximately 138–235 aa (central domain 1 (cd1)), 236–38 aa (cd2), 377–451 aa (cd3), 462–544 aa (cd4), 556–645 (cd5), 668–798 aa (cd6), and 806–873 aa (cd7). A DALI search using predicted structures of those domains indicated their resemblance to pyocin fibers, phage tailspikes, tail fiber proteins, and bacterial cell wall surface anchor family proteins (DALI Z-score about 4–10). Apparently, Ka2 TFP does not have depolymerase activity since all cases of structural similarity were found for the identified carbohydrate-binding domains, but not for the enzymatic domains.

A DALI search, using the AlphaFold model of Ka2 endolysin, revealed significant structural similarity with other phage endolysins (Supplementary File S2). The highest Z-score (24.6) was observed for the muramidase of *Salmonella* phage SPN1S (PDB #4OK7) [69]. Phage Ka2 endolysin was predicted to have a two-domain structural architecture (Supplementary Figure S3a), typical for many endolysins [70]. An HHpred search identified the *Pseudomonas* phage endolysin KTN6 as the closest sequence homolog to the Ka2 endolysin [71]. Notably, the KTN6 endolysin exhibits considerable sequence similarity to the *Salmonella* phage SPN1S muramidase and has experimentally confirmed to have muramidase activity. The superimposition of the AlphaFold-predicted structure of phage Ka2 endolysin with the experimentally determined structure of the *Salmonella* phage SPN1S muramidase (PDB: 4OK7) using PyMOL yielded an RMSD of 1.6 Å. This structural alignment indicates that Glu51 and Glu60 likely represent the catalytic residues of phage Ka2 endolysin (Supplementary Figure S3b). Unlike most gram-negative bacterial endolysins, the experimentally determined structure of the SPN1S endolysin and the AlphaFold-predicted structure of the phage Ka2 endolysin both display a two-domain architecture with a distinct concave groove between the domains [69]. In contrast to our findings about the endolysin, searches using DALI and HHpred failed to identify close structural homologs of the Ka2 holin in other phages. However, a BLAST search revealed sequence homologs of the Ka2 holin among holins of other *Pbunavirus* phages.

### 3.3. Synergistic Effects of Ka2 with Antimicrobials

Since in several works, the synergistic effects of phages with antimicrobials have been demonstrated, the effect of the combined use of Ka2 with various antimicrobials was investigated by evaluating the MIC of drugs in the presence or absence of *Pseudomonas* phage Ka2. On *P. aeruginosa* PAO1, the presence of the phage increased the efficacy of aminoglycosides (amikacin and gentamicin) by four-fold and had no effect on ciprofloxacin, while the MIC of cefepime and colistin were reduced by 8- and 32-fold, respectively (Table 3). On clinical isolates, Ka2 was also able to increase the effective concentrations of antimicrobials against sensitive and tolerant bacteria (Table 4). Thus, in the presence of the phage, the MIC values of cefepime decreased by 2–8 times, and the MICs of colistin and ciprofloxacin were reduced by up to 16 times (for the sensitive isolates). For aminoglycosides, in half of the strains, no effect was observed, and others became 4–8-fold more sensitive to antimicrobials.

### 3.4. Antibiofilm Activity of Pseudomonas Phage Ka2

**Figure 7:**
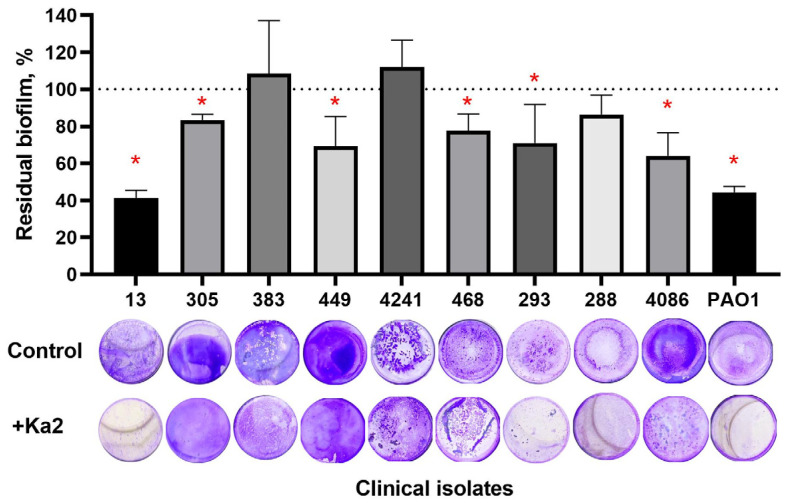
Biofilm destruction with *Pseudomonas* phage Ka2. The 48 h old biofilms of *P. aeruginosa* PAO1 and clinical isolates were treated for 24 h with Ka2 (10^6^ PFUs/mL), and the residual biofilm was evaluated with crystal violet staining. The residual biofilm was calculated as a ratio of the staining intensity of treated and untreated biofilms for each strain separately, considering the untreated biofilm as 100%. Averages and SDs are shown. The significance of differences was assessed using the Kruskal–Wallis test. * *p* < 0.05.

**Figure 8:**
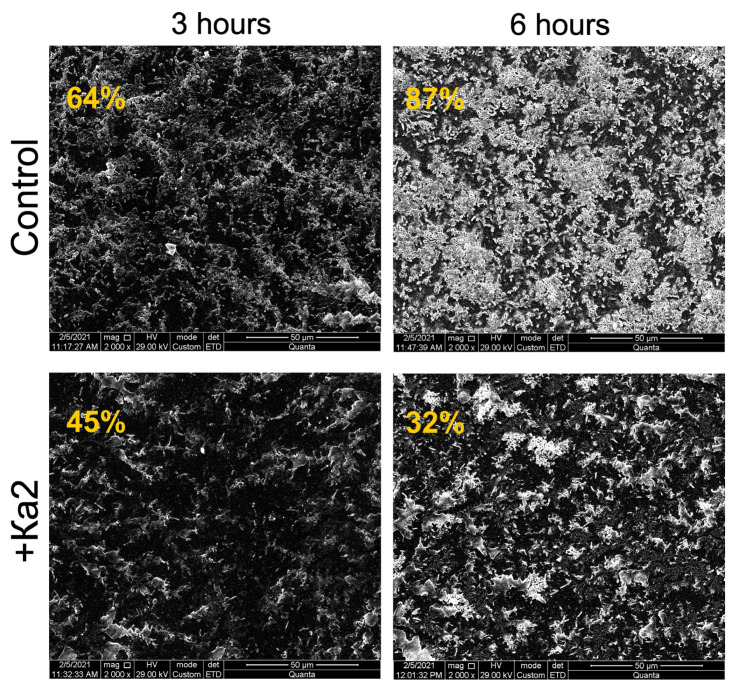
Scanning electron microscopy of the 48 h old biofilms of *P. aeruginosa* PAO1 treated for 3 and 6 h with *Pseudomonas* phage Ka2 (10^6^ PFUs/mL). The percentages show the fraction of the image area covered with the biofilm, calculated using BioFilmAnalyzer software and estimated as the relative fraction of pixels over the threshold of 50 compared to the total image area in pixels. The scale bars indicate 50 μm.

The antibiofilm activity of *Pseudomonas* phage Ka2 has been tested on the host strain *P. aeruginosa* PAO1 and several clinical isolates that are sensitive to the phage and that exhibit various patterns of resistance to antimicrobials (see Table 1 for details). The 48 h old biofilms were treated for 24 h with BM broth supplemented with Ka2 (final concentration of 10^6^ PFUs/mL), and then, the residual biofilm was evaluated with crystal violet staining (Figure 7). The biofilm of the host strain PAO1 was decreased by 50% compared to the initial density, and the reduction was statistically significant compared to the control. The scanning electron microscopy confirmed the disruption of the biofilm (Figure 8): after 3 and 6 h of the treatment, the area covered with biofilm on the plate surface had reduced by 40 and 50%, respectively. The biofilms of isolates 13, 305, 449, 468, 293, and 4087 were reduced by 20–30%. While isolates 383, 4241, and 288 demonstrated sensitivity to Ka2 on the lawn, no decrease in their biofilm biomass could be observed, apparently suggesting that the sensitivity of isolates to the phage does not correlate with the ability of the latter to lyse the biofilm.

Since biofilm destruction should increase bacterial susceptibility to antimicrobials, the effect of the combined use of Ka2 with amikacin, ciprofloxacin, and cefepime for the treatment of biofilms was investigated. The 48 h old biofilms of *P. aeruginosa* PAO1 were grown in 24-well plates, and after gentle washing with sterile PBS, incubation was performed for the next 24 h in fresh BM broth supplemented with antimicrobials at concentrations of 0–256 µg/mL and phage Ka2, as indicated (final concentration of 10^6^ PFUs/mL). Then, the wells were washed, and cell viability was evaluated with CFU count by using the drop plate assay, as described in the Materials and Methods section.

**Figure 9:**
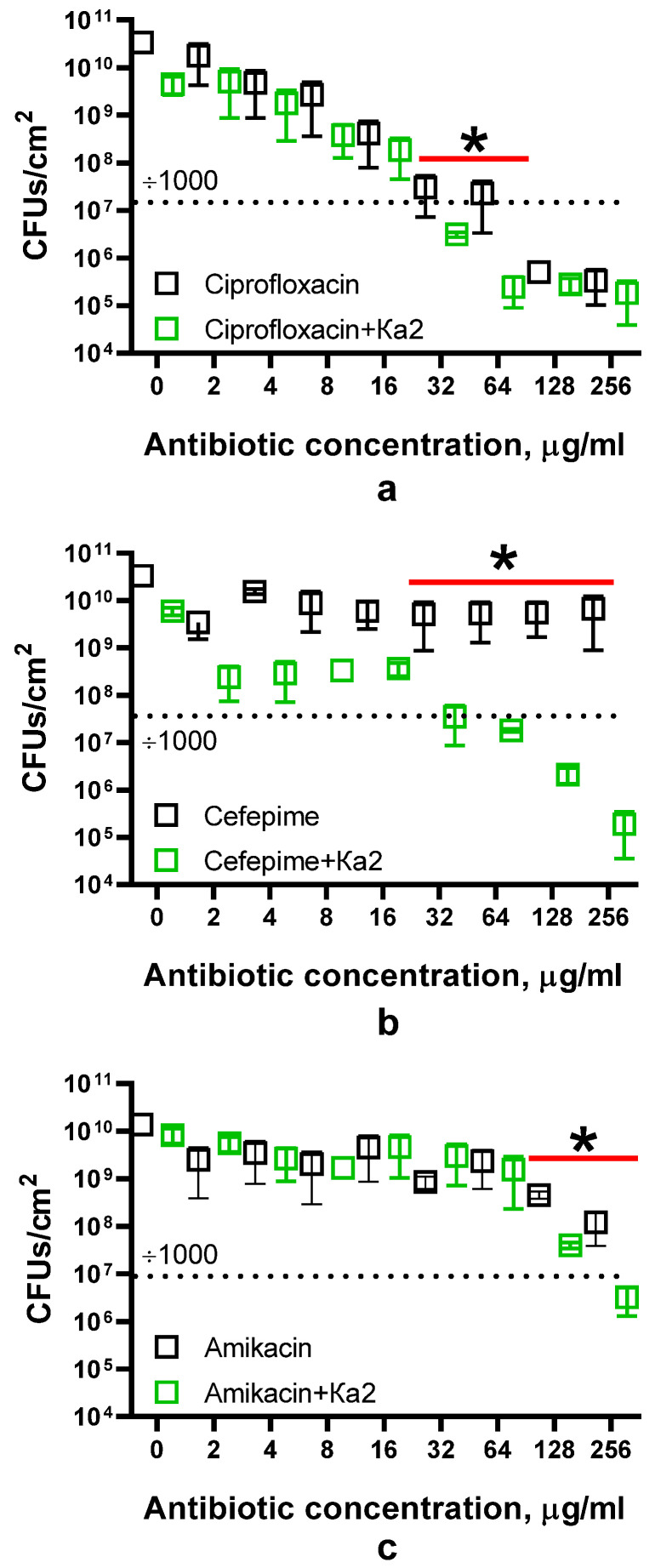
Impact of *Pseudomonas* phage Ka2 on the efficiency of antimicrobials against *P. aeruginosa* biofilms. The 48 h old biofilms of *P. aeruginosa* PAO1 were treated for 24 h with (**a**) ciprofloxacin, (**b**) cefepime, or (**c**) amikacin in the absence (black) or presence (green) of *Pseudomonas* phage Ka2, and then, the remaining CFUs were counted by using the drop plate assay. For CFUs, median and IQR are given. The significance of differences was assessed using the Kruskal–Wallis test with the Holm–Sidak correction. * *p* < 0.05.

On a *P. aeruginosa* PAO1, in the absence of the phage, only ciprofloxacin was able to reduce the number of CFUs by three orders of magnitude at concentrations below 256 µg/mL, and the presence of *Pseudomonas* phage Ka2 did not affect concentration-dependent cell death (Figure 9a). Nevertheless, a slight improvement in efficiency could be observed at 32 and 64 µg/mL of ciprofloxacin. By contrast, no effect of solely cefepime on biofilm-embedded cells could be observed, while in the presence of Ka2, a 3-log decrease in viable cells could be achieved at a concentration of 32 µg/mL (Figure 9b). Amikacin also demonstrated low efficiency against biofilm-embedded cells and reduced the number of CFUs by two orders of magnitude at 128 µg/mL (Figure 9c). In the presence of Ka2, the same reduction in viable cell count could be observed at two-fold lower concentrations of antimicrobials, suggesting a rather additive effect, while up to a four-log reduction in the number of CFUs could be observed at 256 µg/mL of amikacin. On the other hand, the treatment with solely *Pseudomonas* phage Ka2 led to a reduction in CFUs in biofilms only by a factor of 10 (see point 0), suggesting relatively low efficiency of the phage alone when treating the biofilm.

**Figure 10:**
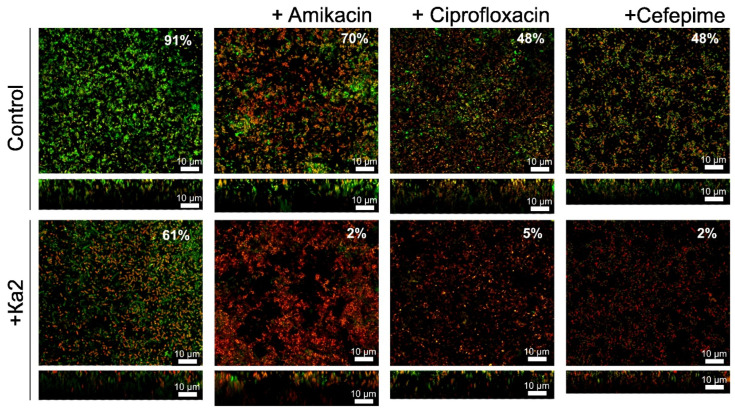
CLSM analysis of the viability of *P. aeruginosa* PAO1 in 48 h old biofilms treated for 24 h with amikacin (256 µg/mL), ciprofloxacin (64 µg/mL), or cefepime (64 µg/mL), as indicated in the absence (upper lane) or presence (lower lane) of *Pseudomonas* phage Ka2. Cells were stained with DioC6 (green: viable) and PI (red: non-viable). The percentages show the fraction of viable cells calculated using BioFilmAnalyzer software and estimated as the relative fraction of the green cells among all cells. The scale bars indicate 10 μm.

The increase in antimicrobial efficiency against biofilm-embedded cells in the presence of Ka2 was also characterized by CLSM. The biofilms were prepared on cell imaging slides and treated for 24 h with amikacin (256 µg/mL), ciprofloxacin, or cefepime (64 µg/mL) solely (control) or in the presence of phage Ka2 (10^6^ PFUs/mL). As can be seen from Figure 10, treatment with either antimicrobials or the phage provides only a two-fold decrease in apparent viable (green stained) cells. When used in combination with Ka2, almost full death of cells could be observed, which fits well with the CFU count data.

The efficiency of antimicrobials against biofilm-embedded cells of *P. aeruginosa* clinical isolates was also investigated by an evaluation of cell viability by using the AlamarBlue test in wells treated with antimicrobials in the presence or absence of the phage Ka2 (Table 5). In combination with the phage, the complete death of cells (biofilm eradication) was observed at antimicrobial concentrations 4–16 times lower compared to solely drugs for isolates 13, 383, 468, and 305 when treated with amikacin and for the *P. aeruginosa* PAO1 strain and isolates 383, 468, and 305 when treated with colistin. A 4-fold reduction in the BEC of cefepime was observed on isolates 383, 449, and 4241, while a 32-fold reduction in the BEC of cefepime was achieved for *P. aeruginosa* PAO1. The activity of ciprofloxacin against the biofilms of isolates was not potentiated by the presence of the phage, while the cells of the PAO1 strain became more susceptible according to the CFU count and CLSM data.

## 4. Discussion

Here, we report a new *Pseudomonas* phage Ka2 of the *Pbunavirus* genus isolated from a tributary stream of Lake Baikal, close to Slyudyanka town. The viruses of this genus (previously known as PB1 viruses) are widely spread in nature and are generally characterized by a conservative genome and excellent lytic properties [28,29,30,31,32,33,72].

Genomic analysis, phylogenetic analysis, and intergenomic similarity calculations indicated that phage Ka2 belongs to the myophage group, which also includes the *Pseudomonas* phage KTN6, a lytic phage that infects *P. aeruginosa* [71]. An endolysin of phage KTN6, similar to the putative endolysin of phage Ka2, was studied and demonstrated muramidase activity. Since the phage endolysins are widely being explored as an alternative to antibiotics, Ka2 could be a putative source for the new antimicrobial enzyme [71]. The Ka2 phage adsorption apparatus includes tail fibers that appear to have a complex structural architecture but are generally reminiscent of the receptor-binding proteins (RBPs) of other phages and pyocins. Several domains appear to be structurally similar to the carbohydrate-binding domains of other RBPs. This architecture of the Ka2 tail fiber proteins may contribute to the efficiency of phage adsorption or to extending the host range of the phage. Indeed, Ka2 was able to lyse at least 21 of 30 clinical isolates of *P. aeruginosa*, including six MDR isolates, while many clinical isolates of *P. aeruginosa* have phage defense systems and up to 68% are not infected by phages [73].

Biofilm formation by *P. aeruginosa* significantly decreases the efficiency of treatment of diseases caused by this microorganism and is a challenging problem in infectious medicine. Therefore, in recent years, a number of new alternative treatment approaches have been developed for targeting *P. aeruginosa* biofilm-associated infections, including antimicrobial peptides, quorum-quenchers, photodynamic therapies, and bacteriophage therapies [15]. Among nine randomly chosen strains sensitive to the phage, Ka2 was able to destroy the biofilm matrix of six strains (Figure 7). Furthermore, the treatment of *P. aeruginosa* biofilms with solely the phage did not lead to its full eradication, although up to a 50% decrease in the biomass was observed. As can be seen from Figure 9 (point 0), in *P. aeruginosa* PAO1 biofilms treated with Ka2 in the absence of antimicrobials, the CFU count decreased only by ten-fold, which also fits with the presence of viable cells on CLSM images of Ka2-treated wells in Figure 10. It is known that phages destroy *P. aeruginosa* biofilms either by inducing the synthesis of their own hydrolytic enzymes (polysaccharide depolymerases [22,74] or endolysins hydrolyzing peptidoglycan [75]) or by inducing the production of bacterial enzymes, like exopolysaccharide hydrolase PelA and PslG, thereby damaging the biofilm of *P. aeruginosa* [76]. Since the biofilms of three isolates (383, 4241, and 288) sensitive to Ka2 on the lawn were not destroyed during the infection, it is more likely that Ka2 implements the biofilm-destroying process via the second scenario, i.e., by inducing the expression of cellular own hydrolytic enzymes, the activity of which can differ in clinical isolates. Nevertheless, the exact mechanisms leading to a reduction in the *P. aeruginosa* biofilms treated with Ka2 remain speculative and require further investigation.

Thus, the obvious disadvantage of Ka2 for use in clinical practice could be its relatively weak lytic ability, despite the broad range of clinical strains that can be infected. These facts clearly suggest that a combined treatment with both phages and conventional antimicrobials is required to achieve the deep eradication of both planktonic and biofilm-embedded cells, as has been discussed previously [39]. Indeed, Ka2 demonstrates the ability to increase the efficiency of conventional antimicrobials. In liquid culture, the MICs of antibiotics on both *P. aeruginosa* PAO1 and clinical isolates were generally reduced by 2–8 times, while the MIC of colistin was reduced by up to 16–32 times. On biofilms, despite various levels of their destruction, the complete death of cells was observed at 8–16-fold lower concentrations of amikacin and colistin compared to those of solely antimicrobials (Table 4). The reason for variations in the potentiation level of different antimicrobials also remains unclear, while the effect of the stoichiometry of the phage–antibiotic concentrations, host microenvironment, and the class of antibiotic has been previously reported [77]. Also, the permeability of bacterial biofilms differs for various antimicrobials [78,79]. Thus, aminoglycosides and cephalosporines are apparently detained by the biofilm matrix, and its phage-driven destruction could facilitate the penetration of these antimicrobials into cells. On the other hand, ciprofloxacin penetrates readily through the biofilm matrix, and the biofilm’s integrity loss does not significantly affect the efficiency of the antimicrobial. This suggestion fits with observations that the biofilm’s destruction level (Figure 7) only partially correlates with the decrease in the effective concentration of antimicrobials (Table 4). Thus, the best potentiation of drugs was observed for PAO1 and isolates 305 and 383, while the best biofilm destruction with solely the phage was observed for PAO1 and isolates 305, 449, 468, and 293, and no decrease in biofilm biomass was documented for isolates 383 and 4241. Nevertheless, these data clearly demonstrate that the combined use of Ka2 phage and antimicrobials could be a helpful tool for biofilm treatment, although not all isolates will be sensitive to the given phage; a preliminary search of appropriate phages for efficient targeting of the biofilm is required.

## 5. Conclusions

Taken together, our data demonstrate the potential of *Pseudomonas* phage Ka2 to infect a broad range of *P. aeruginosa* clinical isolates and potentiate antimicrobials against both planktonic and biofilm-associated cells. The fact that the *Pbunavirus* phages that include the closest relatives of Ka2 are obligatory lytic [80,81,82] and that the Ka2 genome lacks both mobile elements and genes of integrase, transposase, and recombinase, as well as regulators of lysogeny, suggest that Ka2 phage is a promising candidate for use as a therapeutic agent for combined therapy, which fits with earlier opinions about the efficiency of phages of the *Pbunavirus* genus [83].

## Figures and Tables

**Table 1:** Sensitivity of *Pseudomonas aeruginosa* clinical isolates to antimicrobials and bacteriophage *Pseudomonas* phage Ka2.

*P. aeruginosa* Strain and Clinical Isolate	Source, Date of Isolation	Antimicrobial Resistance, Where «R»—Resistance and «I»—Susceptible at Increased Exposure	Sensitivity to Bacteriophage (++++ Pronounced Lysis, +++ Excellent Lysis, ++ Good Lysis, + Weak Lysis, and − No Lysis Observed)
PAO1	The typical strain	-	++++
383 (MDR)	Pharynx, 1.11.2023	Azl-«R», Az-«R», G-«R», L-«R», P-«R», Caz-«R»	++++
13 (MDR)	Pharynx, 20.10.2023	Azl-«R», Az-«R», G-«R», L-«R», P-«R», Caz-«R»	++++
88 (MDR)	Pharynx, 14.09.2023	Azl-«R», Az-«R», G-«R», L-«R», P-«R», Caz-«R»	++++
465 (MDR)	Nasal cavity, 1.11.2023	Azl-«R», Az-«R», L-«R», P-«R», T-«R», Caz-«R»	++++
115 (MDR)	Pharynx, 1.11.2023	Azl-«R», Az-«R», G-«R», L-«R», P-«R», Caz-«R»	++++
468 (MDR)	Pharynx, 14.09.2023	Azl-«R», Az-«R», G-«R», L-«R», P-«R», T-«R», Caz-«R»	++++
400 (MDR)	Pharynx, 27.09.2023	Azl-«R», G-«R», L-«R», P-«R», T-«R», Caz-«R»	−
99 (MDR)	Pharynx, 20.10.2023	Azl-«R», G-«R», L-«R», P-«R», T-«R», Caz-«R»	−
176 (MDR)	Human feces with dysbacteriosis, 27.09.2023	Azl-«R», L-«R», P-«R», T-«R», Caz-«R»	−
1475	Ear canal, 7.04.2022	Az-«I», G-«I», L-«I»	++++
293	Pharynx, 7.04.2022	Azl-«R», Az-«R», G-«R», P-«R», Caz-«R»	++++
288	Ear canal, 7.04.2022	Azl-«R», Az-«I», G-«R», P-«R», Caz-«R»	++++
410	Pharynx, 7.04.2022	Az-«R», G-«R», P-«R», Caz-«R»	++++
206	Pharynx, 27.09.2023	Azl -«R», Az-«R», G-«R», P-«R», T-«R», Caz-«R»	++++
305	Pharynx, 7.04.2022	A-«I», Az-«R», G-«I», P-«R», T-«I», Caz-«R»	++++
4086	Ear canal, 5.09.2022	Azl-«R», Az-«R», G-«I», L-«I», P-«R», Caz-«R»	++++
286	Pharynx, 1.11.2023	Azl-«R», Az-«R», P-«R», Caz-«R»	++++
398	Pharynx, 7.04.2022	Azl-«R», Az-«R»	++++
4241	Nail plates of brushes, 5.09.2022	Azl-«R», Az-«R», G-«R», L-«I», P-«R», Caz-«R»	+++
449	Pharynx, 7.04.2022	Azl-«R», Az-«R», Caz-«R», C-«I»	+++
3101	Eye conjunctiva, 5.09.2022	Azl-«R», Az-«R», L-«I», P-«R», Caz-«R»	++
347	Pharynx, 1.11.2023	Azl-«R», Az-«R», G-«R», P-«R», Caz-«R»	+
2806	Nail plates of brushes, 5.09.2022	Azl-«R», Az-«R», G-«R», L-«I», P-«R», T-«I», Caz-«R»	+
639	Nasal cavity, 14.09.2023	Azl-«R», Az-«R», L-«R», P-«R», Caz-«R»	+
278	Pharynx, 7.04.2022	Az-«R», G-«R», P-«R», Caz-«R»	−
443	Pharynx, 7.04.2022	A-«I», Azl-«R», Az-«R», M-«R», P-«R», C-«R»	−
185	Pharynx, 20.10.2023	Azl-«R», L-«R», P-«R», Caz-«R»	−
250	Pharynx, 7.04.2022	Azl-«R», Az-«R», L-«I», P-«R», Caz-«R»	−
369	Pharynx, 27.09.2023	Azl-«R», G-«R», P-«R», Caz-«R»	−
458	Pharynx, 7.04.2022	A-«I», G-«I»	−

Symbols: Amikacin: A, Azlocillin: Azl, Aztreonam: Az, Gentamicin: G, Levofloxacin: L, Meropenem: M, Piperacillin: P, Tobramycin: T, Ceftazidim: Caz, and Ciprofloxacin: C.

**Table 2:** Comparison of *Pseudomonas* phage Ka2 phage genome to annotated bacteriophage genomes using the BLAST algorithm.

Phage	Genome Size, bp	Coverage	Identity	Accession Number GenBank
*Pseudomonas* phage vB_PaeM_FBPa50	66,569	98%	98%	ON375838.1
*Pseudomonas* phage S50 DNA	64,018	99%	97%	LC472884.1
*Pseudomonas* phage LMA2	64,015	98%	98%	FM201282.1
*Pseudomonas* phage Kara-mokiny kep-wari Wadjak 9	63,994	98%	98%	OP310975.1
*Pseudomonas* phage vB_PaeM_CEB_DP1	63,926	98%	98%	NC_041870.1
*Pseudomonas* phage vB_PaeM_PAO1_Ab27	63,719	99%	97%	LN610579.1
*Pseudomonas* phage Epa21	63,426	98%	97%	MT118298.1
*Pseudomonas* phage shane	63,376	98%	98%	MT119368.1
*Pseudomonas* phage willy	62,711	98%	98%	MT133562.1
*Pseudomonas* phage PaGU11 DNA	62,669	97%	98%	NC_050145.1
*Pseudomonas* phage chunk	62,657	98%	97%	MT119376.1
*Pseudomonas* phage debbie	62,155	98%	97%	MT119363.1
*Pseudomonas* phage vB_PaeM_V524	62,094	97%	98%	MW595221.1
*Pseudomonas* phage S12-3 DNA	61,906	98%	98%	LC472883.1
*Pseudomonas* phage S12-1 DNA	61,906	98%	98%	LC102730.1
*Pseudomonas* phage PA_LZ01	60,672	97%	97%	OM953790.1
*Pseudomonas* phage Epa20	60,152	99%	97%	MT118297.1
*Pseudomonas* phage phiKTN6	58,523	97%	98%	NC_041865.1
*Pseudomonas* phage vB_PaeM_E217	58,519	97%	98%	NC_042079.1
*Pseudomonas* phage vB_PaeM_FBPa12	57,790	98%	98%	ON857930.1

**Table 3:** MIC values of antimicrobials on *P. aeruginosa* PAO1 in the presence or absence of *Pseudomonas* phage Ka2.

Antimicrobials	MIC, µg/mL		MIC Reduction, -Fold
	Control	+Ka2	
Amikacin	1	0.25	4
Gentamicin	0.015	0.0039	4
Colistin	0.25	0.008	32
Ciprofloxacin	0.002	0.004	0.5
Cefepime	8	1	8

**Table 4:** MIC values of antimicrobials on *P. aeruginosa* clinical isolates in the presence or absence of *Pseudomonas* phage Ka2.

Antimicrobials		*P. aeruginosa* Clinical Isolates					
		13	383	468	305	449	4241
Amikacin	Control, MIC, µg/mL	ND	ND	ND	64	ND	ND
	+Ka2, MIC, µg/mL	ND	ND	ND	16	ND	ND
	**MIC reduction, -fold**	**ND**	**ND**	**ND**	**4**	**ND**	**ND**
Gentamicin	Control, MIC, µg/mL	2	2	4	16	ND	16
	+Ka2, MIC, µg/mL	4	2	8	2	ND	4
	**MIC reduction, -fold**	**0.5**	**1**	**0.5**	**8**	**ND**	**4**
Cefepime	Control, MIC, µg/mL	1	4	4	4	2	8
	+Ka2, MIC, µg/mL	0.12	1	0.5	2	1	2
	**MIC reduction, -fold**	**8**	**4**	**8**	**2**	**2**	**4**
Ciprofloxacin	Control, MIC, µg/mL	ND	ND	ND	ND	1	ND
	+Ka2, MIC, µg/mL	ND	ND	ND	ND	0.06	ND
	**MIC reduction, -fold**	**ND**	**ND**	**ND**	**ND**	**16**	**ND**
Colistin	Control, MIC, µg/mL	0.5	1	0.5	0.5	0.5	0.5
	+Ka2, MIC, µg/mL	0.25	0.06	0.06	0.25	0.5	0.12
	**MIC reduction, -fold**	**2**	**16**	**8**	**2**	**1**	**4**

ND—not determined (values are higher than 1024).

**Table 5:** Biofilm-eradicating concentrations (BECs) of antimicrobials on *P. aeruginosa* clinical isolates in the presence or absence of *Pseudomonas* phage Ka2.

Antimicrobials			*P. aeruginosa* Clinical Isolates					
		PAO1	13	383	468	305	449	4241
Amikacin	Control, MIC, µg/mL	8	64	64	16	32	16	8
	+Ka2, MIC, µg/mL	4	16	8	4	2	16	8
	**BEC reduction, -fold**	**2**	**4**	**8**	**4**	**16**	**1**	**1**
Cefepime	Control, MIC, µg/mL	64	8	8	4	4	256	8
	+Ka2, MIC, µg/mL	2	8	2	2	2	64	2
	**BEC reduction, -fold**	**32**	**1**	**4**	**2**	**2**	**4**	**4**
Ciprofloxacin	Control, MIC, µg/mL	2	1	1	1	1	1	1
	+Ka2, MIC, µg/mL	0.5	1	1	1	0.5	0.5	0.5
	**BEC reduction, -fold**	**4**	**1**	**1**	**1**	**2**	**2**	**2**
Colistin	Control, MIC, µg/mL	64	16	64	64	32	64	32
	+Ka2, MIC, µg/mL	8	16	8	16	2	32	16
	**BEC reduction, -fold**	**8**	**1**	**8**	**4**	**16**	**2**	**2**

## Data Availability

Data are present in this study or are available by request.

## Supplementary Materials

The following supporting information can be downloaded at: https://www.mdpi.com/article/10.3390/v17020189/s1, Figure S1: (a) AF model of predicted tail fiber protein (monomer) of phage Ka2 visualized using PyMOL. The rainbow coloring uses a color gradient where the N-terminal end is blue and the C-terminus is red. (b) Quality of structure prediction obtained using AF. The coloring is explained in the legends. Figure S2: (a) AF model of predicted trimeric tail fiber of phage Ka2 (each monomer has a different color) visualized using PyMOL. (b) Quality of structure prediction obtained using AF. The coloring is explained in the legends. Figure S3: (a) AF model of predicted endolysin Ka2 visualized using PyMOL. The rainbow coloring uses a color gradient where the N-terminal end is blue and the C-terminus is red. (b) Superimposition of the AlphaFold-predicted structure of phage Ka2 endolysin with the experimentally determined structure of the *Salmonella* phage SPN1S muramidase (PDB code 4ok7) using PyMOL. The coloring is explained in the legends. The predicted catalytic residues (Glu51 and Glu60) are shown using stick representation. (c) Quality of structure prediction obtained using AF. The coloring is explained in the legends. File S1: DALI search results using the AF model of Ka2 tail fiber. File S2: DALI search results using the AF model of Ka2 endolysin.
