# Supplementary material for: New Bacteriophage Pseudomonas Phage Ka2 from a Tributary Stream of Lake Baikal
Source: Viruses. 2025 Jan 29;17(2):189. doi: 10.3390/v17020189 (PMC11861027; doi:10.3390/v17020189)
Supplement: Supplementary file 1 [file viruses-17-00189-s001.zip › Supplementary figures.pdf]

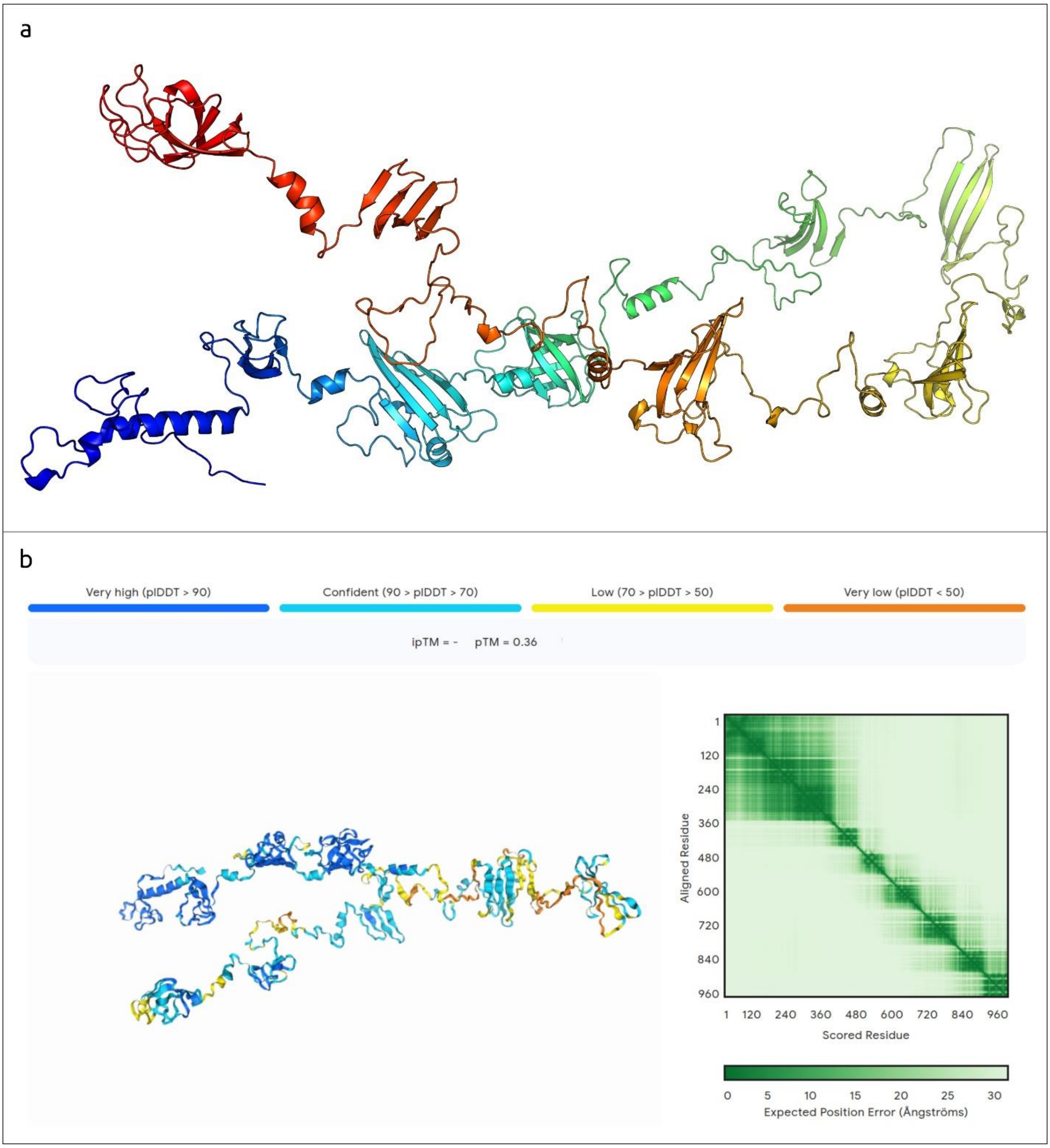

Figure S1: **(a)** AF model of predicted tail fiber protein (monomer) of phage Ka2 visualized using PyMOL. Rainbow coloring uses a color gradient where the N-terminal end is blue and the C-terminus is red. **(b)** Quality of structure prediction obtained using AF. Coloring is explained in legends.

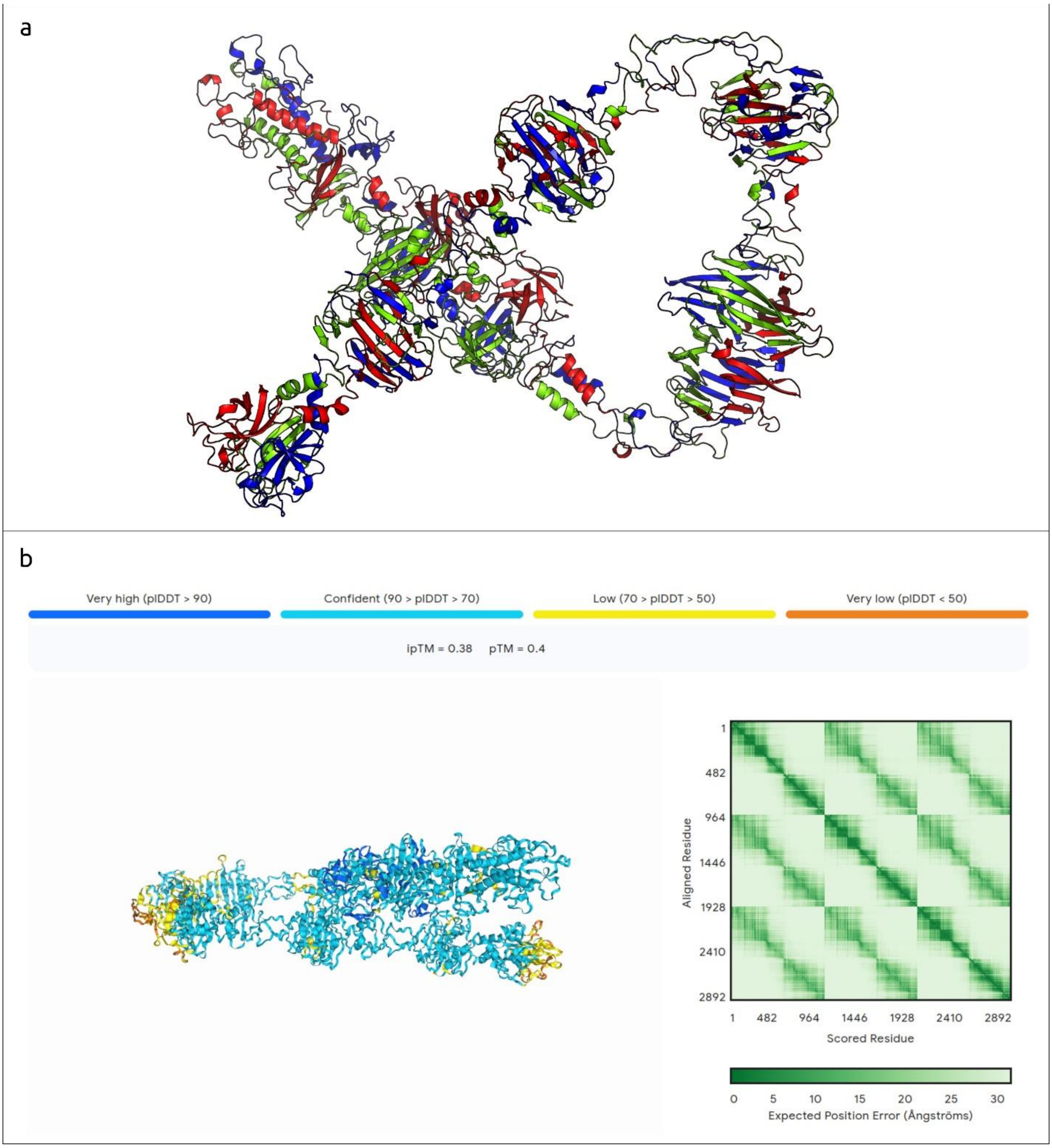

Figure S2: **(a)** AF model of predicted trimeric tail fiber of phage Ka2 (each monomer has a different color) visualized using PyMOL. **(b)** Quality of structure prediction obtained using AF. Coloring is explained in legends.

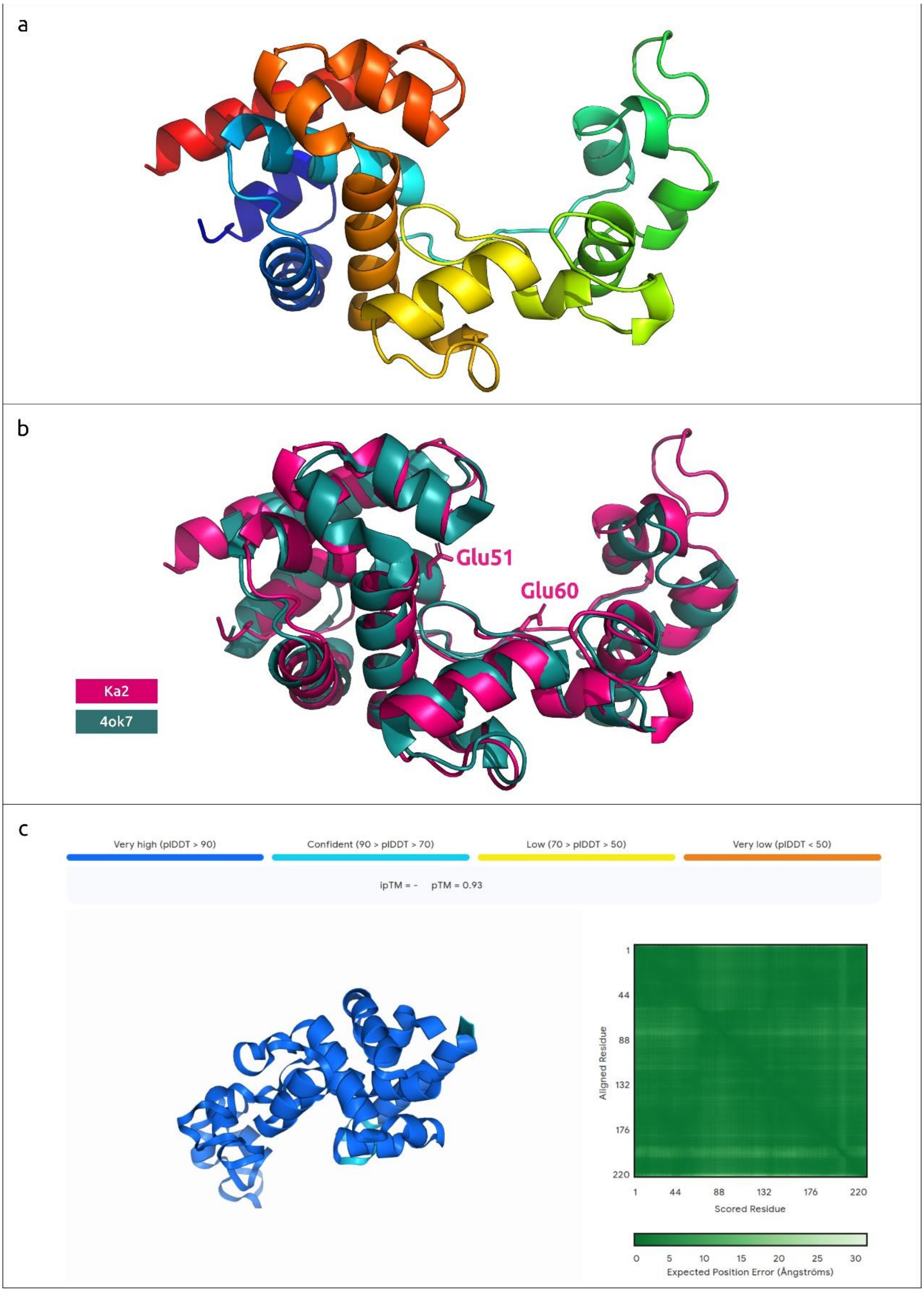

Figure S3: **(a)** AF model of predicted endolysin Ka2 visualized using PyMOL. Rainbow coloring uses a color gradient where the N-terminal end is blue and the C-terminus is red. **(b)** Superimposition of the AlphaFold-predicted structure of phage Ka2 endolysin with the experimentally determined structure of the *Salmonella* phage SPN1S muramidase (PDB code 4ok7) using PyMOL. Coloring is explained in legends. Predicted catalytic residues (Glu51 and Glu60) are shown using stick representation. **(c)** Quality of structure prediction obtained using AF. Coloring is explained in legends.
